# Transcriptomic analysis of cardiac gene expression across the life course in male and female mice

**DOI:** 10.14814/phy2.14940

**Published:** 2021-07-10

**Authors:** Aykhan Yusifov, Vikram E. Chhatre, Eva K. Koplin, Cortney E. Wilson, Emily E. Schmitt, Kathleen C. Woulfe, Danielle R. Bruns

**Affiliations:** ^1^ Kinesiology and Health University of Wyoming Laramie WY USA; ^2^ WY INBRE Bioinformatics/Data Science Core Laramie WY USA; ^3^ Division of Cardiology University of Colorado‐Denver Aurora CO USA; ^4^ Division of Geriatric Medicine University of Colorado‐Denver Aurora CO USA

**Keywords:** aging, cardiac, life course, Omics, RNA‐seq, sex differences

## Abstract

Risk for heart disease increases with advanced age and differs between sexes, with females generally protected from heart disease until menopause. Despite these epidemiological observations, the molecular mechanisms that underlie sex‐specific differences in cardiac function have not been fully described. We used high throughput transcriptomics in juvenile (5 weeks), adult (4–6 months), and aged (18 months) male and female mice to understand how cardiac gene expression changes across the life course and by sex. While male gene expression profiles differed between juvenile‐adult and juvenile‐aged (254 and 518 genes, respectively), we found no significant differences in adult‐aged gene expression. Females had distinct gene expression changes across the life course with 1835 genes in juvenile‐adult and 1328 in adult‐aged. Analysis of differentially expressed genes (DEGs) suggests that juvenile to adulthood genes were clustered in cell cycle and development‐related pathways in contrast to adulthood‐aged which were characterized by immune‐and inflammation‐related pathways. Analysis of sex differences within each age suggests that juvenile and aged cardiac transcriptomes are different between males and females, with significantly fewer DEGs identified in adult males and females. Interestingly, the male–female differences in early age were distinct from those in advanced age. These findings are in contrast to expected sex differences historically attributed to estrogen and could not be explained by estrogen‐direct mechanisms alone as evidenced by juvenile sexual immaturity and reproductive incompetence in the aged mice. Together, distinct trajectories in cardiac transcriptomic profiles highlight fundamental sex differences across the life course and demonstrate the need for the consideration of age and sex as biological variables in heart disease.

## Graphical abstract

Cardiac gene expression changes across the life course in a sex specific manner. These differences in cardiac gene expression by age do not appear to be due to estrogen alone, given significant differences in gene expression juvenile and aged animals.

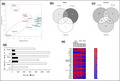

## 1 INTRODUCTION

Advanced age is the most significant risk factor for heart disease, with females generally protected against heart disease until menopause. Despite these epidemiological observations, however, the molecular mechanisms that underlie sex‐specific differences in cardiac function and disease have not been fully described. Identification of the molecular underpinnings of the healthy aging heart and the changes in transcriptomic profiles which occur across the life course will facilitate the identification of distinct risk factors for disease development between men and women. Current literature suggests that cardiac aging is associated with progressive cardiac remodeling and patterns of this remodeling differ between the sexes across the life course (Cheitlin, 2003; Ribera‐Casado, 1999) such as the higher degree of concentric remodeling of the left ventricle (LV) and incidence of diastolic dysfunction in older women (Gori et al., 2014; Kane et al., 2011; Lam et al., 2011). Mechanistically, extracellular matrix (ECM) deposition may contribute to these sex differences, as fibrosis is more pronounced in aging male compared to female hearts (Achkar et al., 2020; Kessler et al., 2019). Historically, sex‐specific gene expression and phenotypic differences have been ascribed to the expression of genes located on sex chromosomes and sex hormones such as estrogen. However, the molecular mechanisms underlying cardiac aging are still not yet clear with respect to biological sex and are likely more complex than estrogen alone, based on observations that sex differences persist post‐menopausally (Gori et al., 2014; Krumholz et al., 1993) or in mice that are reproductively incompetent (Bergmann et al., 1995).

RNA sequencing (RNA‐seq) permits the identification of global differences in gene expression, including differences by age and sex, and has been successfully used to understand pathophysiological changes in the heart (Lai et al., 2019; Yusifov et al., 2021). Previously, we reported age‐related changes in cardiac function and structure in juvenile, adult, and aged mice of both sexes (Yusifov et al., 2021). Here, we aimed to understand changes in cardiac gene expression by sex using high throughput transcriptomics to begin to identify the mechanisms by which cardiac function and structure change across the life course. We hypothesized that gene expression would differ in juvenile, adult, and aged male and female mice, resulting in distinct phenotypes of cardiac aging. Further, we hypothesized that significant sex differences would exist with respect to cardiac gene expression. To test this hypothesis, we performed RNA‐seq in the hearts of male and female C57BL/6 mice at three distinct ages‐ juvenile, adult, and aged to identify sex‐specific differences in cardiac gene expression across the life course.

## 2 MATERIALS AND METHODS

### 2.1 Study design and animals

C57BL/6 mice were purchased from Jackson Laboratories and bred at the University of Wyoming. Aged male and female C57BL/6 mice were donated from the National Institute of Aging Rodent Colony and allowed to acclimate in Laramie Wyoming for 1 week before sacrifice. Analyses were performed at three distinct ages‐ juvenile (5 weeks), adult (4–6 months), and aged (18–20 months). The juvenile age was selected with consideration of the mouse juvenile developmental period ending by 6 weeks. The 18‐month‐old aged cohort demonstrates significant evidence of age‐associated LV hypertrophy and are consistent with publications in aged mice without significant decreases in survival in the basal condition (Turturro et al., 1999). Mice were housed under a 12 h light:dark cycle at 20–23°C in specific pathogen‐free facility, supplied with standard rodent food and water. Following an overnight fast, animals were humanely euthanized with Fatal Plus (390 mg/ml pentobarbital sodium). The whole heart was harvested, followed by dissection of the right ventricle from the LV and septum, which was flash‐frozen in liquid N_2_ and stored at −80°C. All animal procedures were in accordance with the standards set by the Institutional Animal Care and Use Committee at the University of Wyoming.

Female mouse sexual maturity and reproductive competence were assessed by vaginal opening, uterine weight, and estrous cycling. Vaginal openings were assessed daily starting at weaning at 21 days of age and continuing until the vaginal opening appeared (Caligioni, 2009). Uterine weights were collected by the dissection of uteri, collection of weights, and light photography. Estrous cycling was determined by vaginal cytology. Briefly, following three to five washes with phosphate‐buffered saline, a vaginal sample was collected and observed under light microscopy (Caligioni, 2009). In females, wheel running can be used as an indicator to assess estrus cycling (Kent et al., 1991); therefore, for the collection of wheel‐running data, a separate cohort of juvenile, adult, and aged female mice was housed alone with a running wheel for 2 weeks (Columbus Instruments) to be used voluntarily. Each wheel had a magnetic indicator and hall effect sensor connected to a computer interface and recorded wheel revolutions (converted to kilometers). Wheel‐running data were collected daily for 14 days, and mice were routinely checked to ensure the wheel was still functioning properly (Bruns et al., 2020). To validate our indirect measurements of estrogen cycling, we also quantified plasma estradiol in female mice. Briefly, blood samples were collected in EDTA at sacrifice from the chest cavity and centrifuged at 12,000× *g* for 5 min. Estradiol parameter assay was performed following manufacturers’ instruction for 17β‐estradiol content (Estradiol Assay Kit, Abcam).

### 2.2 RNA sequencing

RNA was isolated following standard TRIzol protocols and cleaned using a commercially available kit for genomic DNA depletion (Qiagen Inc). Nine TruSeq RNA libraries from each sex (18 total) were sequenced using the NovaSeq 6000 platform to generate 150bp paired‐end reads. Reads were assessed for quality and contamination using FastQC (Andrews, 2010). Reads were trimmed for adapter contamination using the BBDuk tool (Bushnell, 2014) and for a minimum PHRED‐scaled quality score of 26 using Trimmomatic (Bolger et al., 2014). Library preparation and sequencing were performed by the University of Colorado‐Denver Genomics and Microarray Core. The read data were aligned with the mouse reference genome assembly version 38.97 and genome annotation version 38 from Ensembl using HISAT2 genome aligner (Kim et al., 2019). Transcripts were assembled using StringTie version 1.3.4 (Pertea et al., 2015). Raw counts of identified transcripts and genes were analyzed in EdgeR (Robinson et al., 2010) for differential expression. Counts were normalized and expression dispersion was estimated. Fisher's exact test was used to estimate statistical significance and determine False Discovery Rate (FDR). Gene Set Enrichment Analysis (GSEA) and the Kyoto Encyclopedia of Genes and Genomes (KEGG) analysis were performed to understand the potential molecular mechanisms of Differentially Expressed Genes (DEGs) using the clusterProfiler (version 3.14.3), ggplot2 (version 2_3.3.2), and enrichplot packages (version 1.6.1) in R software (Yu et al., 2012). Bioconductor pathway was used for genome‐wide annotation (org. Mm.eg.db; version 3.10.0). ShinyGO (version 0.61) was used for gene ontology enrichment analysis for clustering DEGs. Significance was set to *p* < 0.05 for GSEA and KEGG analysis. The raw RNA‐seq data have been deposited in the NCBI SRA database under the PRJNA640422 number.

### 2.3 Quantitative RT‐PCR

Standard TRIzol protocol was used to extract RNA from LV tissue and reverse transcribed (High‐capacity RNA‐to‐cDNA kit; Fisher Scientific). Quantitative PCR was run with Power SYBR Green qPCR (Fisher Scientific) and ABI7300 (Fisher Scientific). The expression of target genes was normalized to the expression of 18s ribosomal RNA (Ellefsen et al., 2012). Primer sequences were as follows: Periostin (POSTN) Forward: 5′‐CCATTGGAGGCAAACAACTCC‐3′, Reverse: 5′‐TTGCTTCCTCTCACCATGCA‐3′; Fibronectin (FN1) Forward: 5′‐AGGCAATGGACGCATCAC‐3′, Reverse: 5′‐TTCCTCGGTTGTCCTTCTTG‐3′; Alpha‐1 type I Collagen (COL1A1) Forward: 5′‐GACGCCATCAAGGTCTACTG‐3′, Reverse: 5′‐ACGGGAATCCATCGGTCA‐3′. Results were analyzed by ∆∆Ct with values are presented as fold change from the juvenile group within sex.

### 2.4 Proteomics

Plasma proteomics was performed by the University of Washington Nathan Shock Core. Briefly, protein samples were quantified using a Bradford assay. Samples were then reduced and cysteine blocking and trypsin digestion as previously described (Cilia et al., 2009). Three biological replicates for each age were diluted and prepped (Pino et al., 2020). The samples were acidified to pH 2 with formic acid and desalted using C18 Sep‐Pak cartridges (Waters) according to the instructions from the manufacturer. The peptide solution was then concentrated using an EZ‐2 Plus vacuum centrifuge (Genevac), reconstituted with 0.1% formic acid at a concentration of 1 mg/ml, and stored at −80°C until LC‐MS/MS analysis. Samples were run through mass spectrometry for measuring peptide abundance in plasma. Raw data were refined and quantified by the Skyline software package (MacLean et al., 2010). Quantified data were analyzed by quantification tools in the R platform (version 4.0.3) (De Livera et al., 2018; Leek & Storey, 2007; Ritchie et al., 2015).

### 2.5 Statistics

PCR and plasma estradiol data were assessed by one‐way ANOVA with Tukey's post hoc as warranted. Uterine weights were assessed by Student's *t*‐test. Data were log transformed where data did not meet assumptions for homoscedasticity. Statistical significance was set a priori at *p* < 0.05. For these analyses, SPSS version 25 (IBM Corp.) was used. Data are expressed as mean ± SD.

## 3 RESULTS

### 3.1 Cardiac transcriptomics across the life course

**FIGURE 1:**
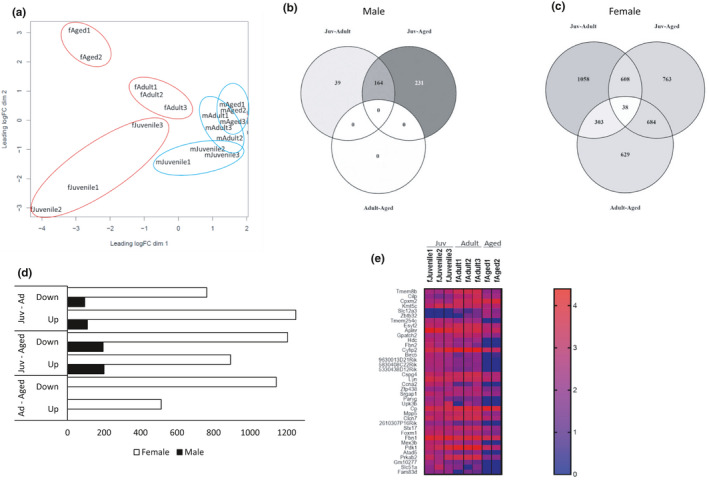
Differentially Expressed Genes (DEGs) across the life course in the heart. (a) PCA plot: juvenile (fJuvenile‐ female juvenile; mJuvenile‐ male juvenile), adult (fAdult‐ female adult; mAdult – male adult), and aged (fAged‐ female aged, mAged‐ male aged); (b) Venn diagram for DEGs in male age comparisons (FDR 5%): Juv‐Adult, adult compared to juvenile as a baseline; Juv‐Aged, aged compared to juvenile as a baseline; adult‐aged, aged compared to adult as a baseline; (c) Venn diagram for DEGs in female age comparisons (FDR 5%): Juv‐Adult, adult compared to juvenile as a baseline; Juv‐Aged, aged compared to juvenile as a baseline; adult‐aged, aged compared to adult as a baseline; (d) Bar graph for up‐ and downregulated DEGs; Juv‐Ad, DEGs from the comparison of adult to juvenile as a baseline; Juv‐Aged, DEGs from aged compared to juvenile as a baseline; Ad‐Aged, DEGs from aged compared to adult as a baseline. (e) Heatmap of 38 overlapping DEGs in females. n = 3 per group; false discovery rate (FDR) <0.05

Gene expression across the life course differed by sex, with females of all ages clustered distinctly from males (Figure 1a). In males, using the pairwise exact test, we identified 203 and 395 DEGs in juvenile‐adult and juvenile‐aged comparisons, respectively. Interestingly, male adult‐aged pairwise comparison produced no DEGs (Figure 1b). Analysis of females produced a more robust list of DEGs, yielding 2013, 2096, and 1656 genes for juvenile‐adult, juvenile‐aged, and adult‐aged (Figure 1c). Comparison of differentially expressed genes across all ages identified 38 DEGs, with the heatmap of these genes demonstrating distinct differences across age (Figure 1e). While the relative number of down and upregulated DEGs was similar for males in juvenile‐adult and juvenile‐aged, females differed in the number of up‐ or downregulated genes by age, with juvenile‐adult demonstrating more upregulated genes, while juvenile‐aged and adult‐aged were more downregulated (Figure 1d).

**FIGURE 2:**
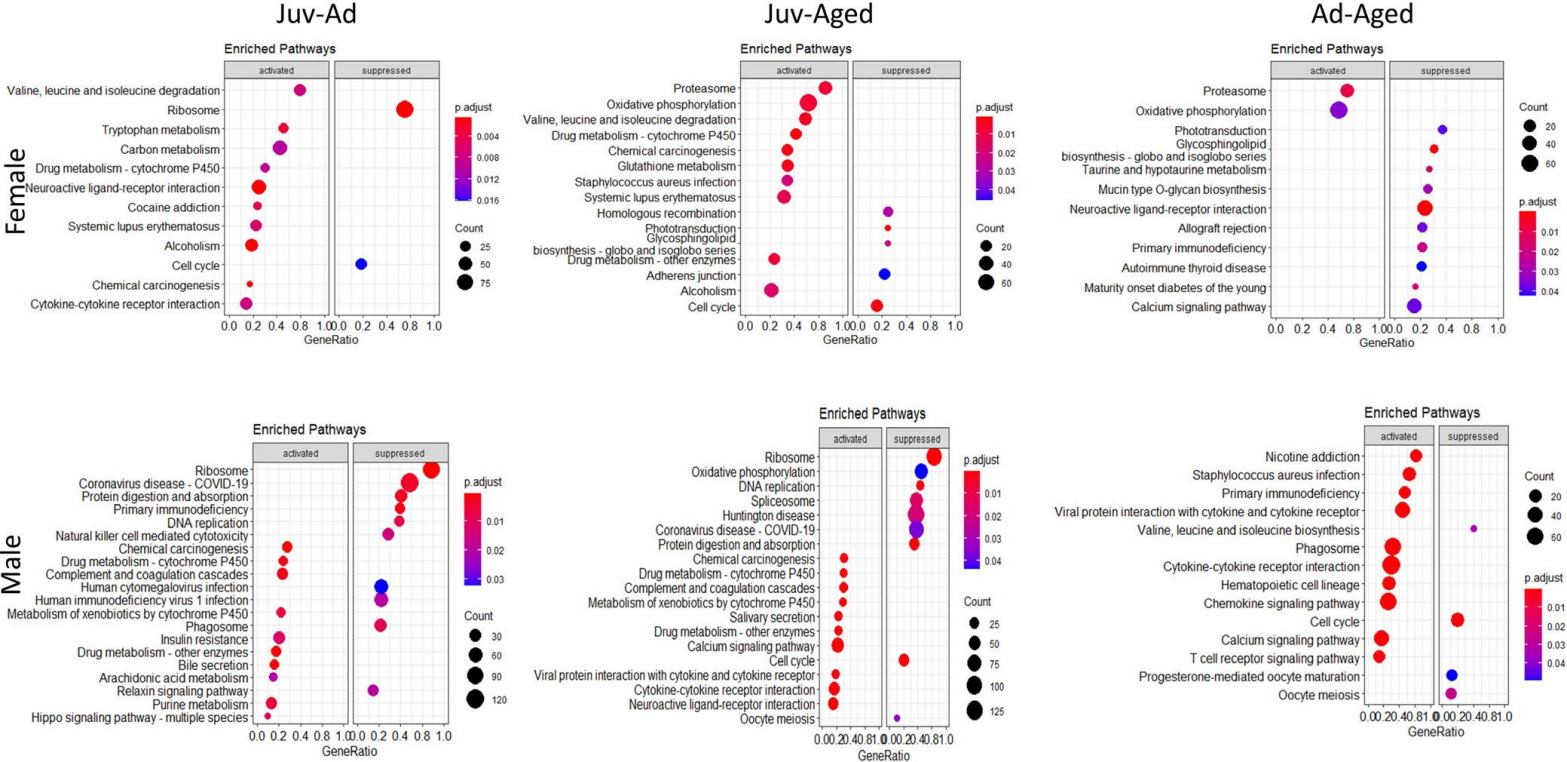
Kyoto Encyclopedia of Genes and Genomes (KEGG) analysis for comparison of transcriptomic profiles of both sexes by age (FDR 5%). Juv‐Ad, enriched pathways from adult compared to juvenile as a baseline; Juv‐Aged, enriched pathways from aged compared to juvenile as a baseline; Ad‐Aged, enriched pathways from aged compared to adult as a baseline

We carried out functional annotation analysis using ShinyGO to investigate the biological relevance of DEGs (Ge et al., 2020). In male mice, genes in juvenile‐adult were clustered in pathways related to ECM organization and intracellular signaling pathways such as ECM‐receptor interaction, focal adhesion, and PI3K‐Akt signaling (Table 1). By Kyoto Encyclopedia of Genes and Genomes (KEGG) analysis, we found a significant enrichment of cell cycle‐related pathways and immune response pathways (Figure 2). The juvenile‐aged gene enrichment was similar to juvenile‐adult, with ECM‐receptor interaction, cell adhesion molecules, and tight junction pathways being the top pathways enriched. Since we did not identify any DEGs in adult‐aged, clustering of genes was not applicable for this comparison. However, we used KEGG analysis for investigating general activation versus suppression patterns of genes between these ages and observed that most of the pathways were immune response related and hinted to the activation of inflammation as aging occurs (Figure 2).

**TABLE 1:** Gene Ontology Enrichment Analysis for DEGs across the life course by sex (FDR 5%)

	Males				Females			
	Enrichment FDR	Genes in list	Total genes	Functional Category	Enrichment FDR	Genes in list	Total genes	Functional Category
Juvenile‐Adult	1.40E‐10	13	90	Protein digestion and absorption	7.00E‐09	36	119	Carbon metabolism
	2.70E‐06	9	83	ECM‐receptor interaction	4.60E‐07	21	55	Valine, leucine and isoleucine degradation
	7.60E‐06	12	198	Focal adhesion	1.10E‐06	175	1301	Metabolic pathways
	4.70E‐04	9	167	Phagosome	6.50E‐05	13	31	Propanoate metabolism
	6.00E‐03	6	103	Amoebiasis	8.10E‐05	13	32	Citrate cycle (TCA cycle)
	1.20E‐02	10	353	PI3K‐Akt signaling pathway	2.60E‐04	17	57	Fatty acid metabolism
	1.20E‐02	6	136	Apoptosis	5.40E‐04	13	38	Pyruvate metabolism
	1.20E‐02	5	86	Gap junction	2.10E‐03	11	32	Beta‐Alanine metabolism
	1.20E‐02	6	129	Relaxin signaling pathway	3.10E‐03	20	90	Protein digestion and absorption
	1.20E‐02	7	171	Alzheimer disease	9.10E‐03	13	50	Fatt acid degradation
Juvenile‐Aged	9.20E‐08	14	90	Protein digestion and absorption	9.00E‐05	24	83	ECM‐receptor interaction
	1.60E‐04	10	83	ECM‐receptor interaction	2.00E‐03	18	64	Glutathione metabolism
	2.90E‐03	14	227	Thermogenesis	3.40E‐02	13	50	Fatty acid degradation
	6.40E‐03	17	348	Human papillomavirus infection	3.40E‐02	14	55	Valine, leucine and isoleucine degradation
	7.50E‐03	11	171	Alzheimer disease	3.40E‐02	14	58	Lysine degradation
	1.20E‐02	7	78	Cardiac muscle contraction	3.40E‐02	19	92	Small cell lung cancer
	1.20E‐02	10	159	Cell adhesion molecules (CAMs)	3.60E‐02	10	35	Fructose and mannose metabolism
	1.30E‐02	10	167	Phagosome	3.60E‐02	20	101	AGE‐RAGE signaling pathways in diabetic complications
	1.30E‐02	11	198	Focal adhesion	3.80E‐02	15	68	Drug metabolism
	1.30E‐02	10	166	Tight junction	3.80E‐02	22	119	Carbon metabolism
Adult‐Aged	NO DEGs				8.40E‐20	48	129	Ribosome
					3.00E‐04	26	131	Oxidative phosphorylation
					3.00E‐04	37	227	Thermogenesis
					5.80E‐04	26	140	Parkinson disease
					9.10E‐04	29	171	Alzheimer disease
					3.00E‐03	25	148	Non‐alcoholic fatty liver disease (NAFLD)
					1.00E‐01	25	189	Huntington disease
					1.80E‐01	12	72	Adherens junction
					2.70E‐01	27	231	Ras signaling pathway
					2.70E‐01	12	78	Cardiac muscle contraction

In females, juvenile‐adult DEGs were categorized in metabolic pathways (Table 1). Juvenile‐aged DEGs were clustered in cardiac remodeling related, metabolic pathways, ECM‐receptor interaction, and glutathione metabolism. KEGG analysis yielded similar results with suppression of cell cycle pathways, activation of metabolic pathways including glutathione metabolism. Additionally, in both juvenile‐adult and juvenile‐aged comparisons, DEGs clustered in valine, leucine, and isoleucine degradation pathways (Table 1). Female adult‐aged DEGs clustered in immune response pathways along with few cell signaling and ECM organization pathways (Table 1). KEGG analysis produced only two activated pathways: proteasome and oxidative phosphorylation. This analysis also indicated the suppression of significant cardiac‐related pathways, such as taurine and hypotaurine metabolism and calcium signaling.

### 3.2 Expression of ECM‐related genes in the LV across the life course

**FIGURE 3:**
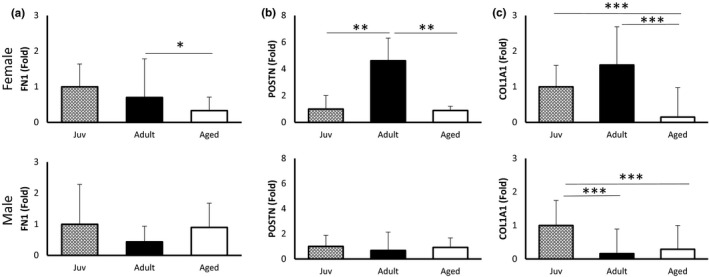
Expression of pro‐fibrotic genes across the life course by sex. (a) Fibronectin (FN1) expression was lower in aged female mice compared to adult, but not different in males. (b) Periostin (POSTN) expression in females was higher in adulthood but lower in aged with no differences observed in males (c) Alpha‐1 type I collagen (COL1A1) expression in females was lower in aged mice compared to adults and juvenile but lower in adults and aged compared to juvenile in males. n = 6 per group. *p<0.05, **p<0.01, ***p<0.001 by one‐way ANOVA

Since we repeatedly observed clustering of DEGs in ECM‐related pathways, we assessed the expression of pro‐fibrotic genes fibronectin (FN1), periostin (POSTN), and collagen (COL1A1) by RT‐PCR in LV tissues of juvenile, adult, and aged male and female mice (Figure 3). Sexual dimorphism was present in the expression of all of these genes across the life course. FN1 expression was not significantly different across the life course in male hearts, but in females FN1 expression was lower in aged hearts compared to adult and juvenile (Figure 3a). POSTN expression was higher in adult female hearts but unchanged in males (Figure 3b). COL1A1 expression, was lower in aged female hearts compared to adult and juvenile. However, in males, lower expression of COL1A1 occurred earlier in adults compared to juveniles, confirming sex‐and age‐specific differences in the RNA‐seq enrichment of ECM‐related genes across the life course.

### 3.3 Cardiac transcriptomics by sex

**FIGURE 4:**
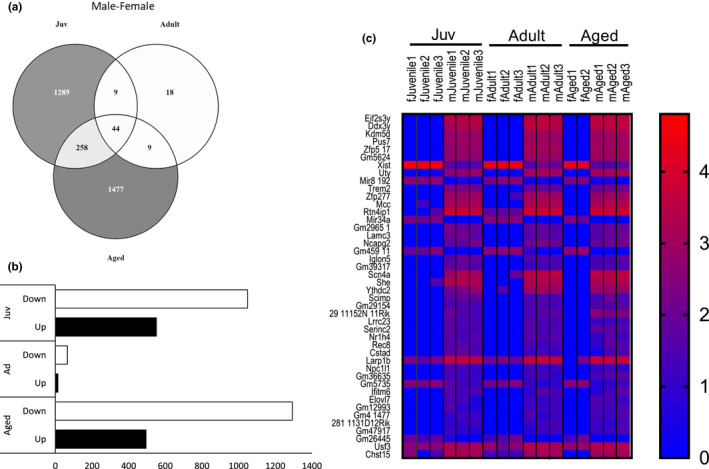
Differentially Expressed Genes (DEGs) by sex across the life course within age. (a) Venn diagram for DEGs from sex comparisons (FDR 5%): Juv‐juvenile female compared to juvenile male as a baseline; adult, adult female compared to adult male as a baseline; aged, aged female compared to aged male as a baseline; (b) Bar graph for up‐ and downregulated DEGs from sex comparisons; Juv, juvenile female compared to juvenile male as a baseline; Ad, adult female compared to adult male as a baseline; Aged, aged female compared to aged male as a baseline. (c) Heatmap of 44 overlapping DEGs across the life course. n = 3 per group; FDR < 0.05

**FIGURE 5:**
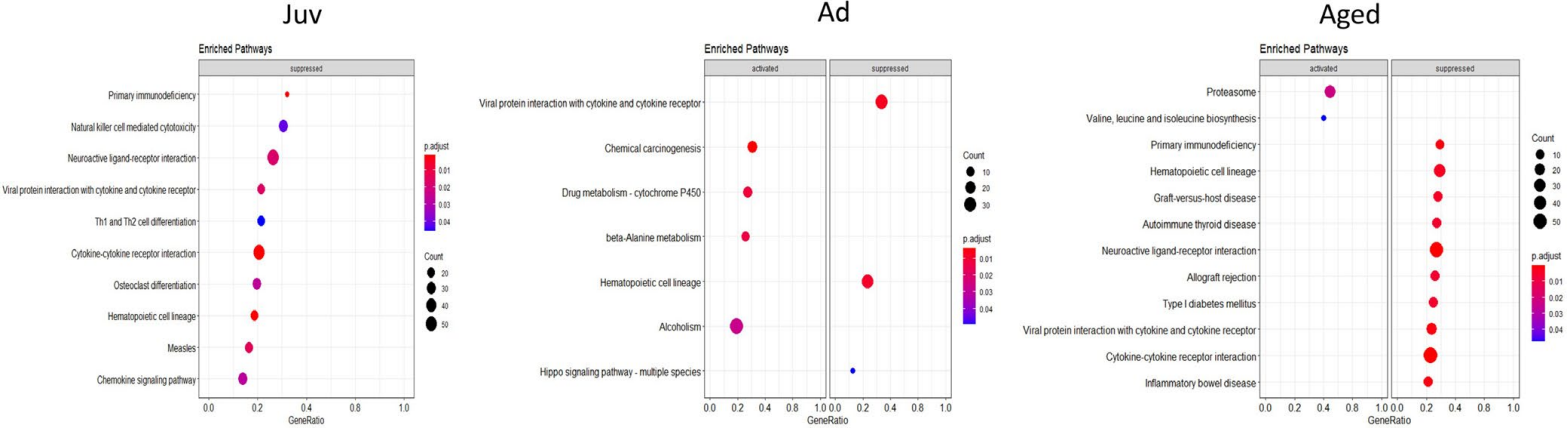
Kyoto Encyclopedia of Genes and Genomes (KEGG) analysis for the sex comparison of transcriptomic profiles within age (FDR 5%). Juv, juvenile female compared to juvenile male as a baseline; Ad, adult female compared to adult male as a baseline; Aged, aged female compared to aged male as a baseline

Next, we compared gene expression profiles between sexes within each age. Pairwise comparisons produced 1600, 80, and 1788 DEGs for juvenile, adult, and aged, respectively (Figure 4a). Of these DEGs, 1049 genes were downregulated and 551 upregulated in juveniles, 66 downregulated and 14 upregulated in adults, and 1293 downregulated and 495 upregulated in aged male‐female comparisons (Figure 4b,c). Interestingly, the few DEGs in the overlap of juvenile and aged comparisons indicated that juvenile transcriptomic sex differences were not the same as aged transcriptomic sex differences. We again carried out functional annotation analysis to identify categories of DEGs for each age. DEGs for juveniles clustered mostly in metabolic pathway functional categories (Table 2). KEGG analysis suggested the suppression of cell differentiation and development (Figure 5). DEGs from aged male‐female were clustered in cell signaling and immune response pathways, with the suppression of these immune response pathways in females compared to males (Figure 5).

**TABLE 2:** Gene Ontology Enrichment Analysis for DEGs between males and females within age (FDR 5%)

	Enrichment FDR	Genes in list	Total genes	Functional Category
Juvenile	7.10E‐04	24	119	Carbon metabolism
	6.10E‐03	15	66	Glycolysis/Gluconeogenesis
	1.90E‐02	9	32	Citrate cylce (TCA cycle)
	1.90E‐02	9	31	Propanoate metabolism
	4.70E‐02	17	103	HIF‐1 signaling pathway
	4.90E‐02	9	38	Pyruvate metabolism
Adult	3.70E‐02	2	36	African trypanosomiasis
	3.70E‐02	2	47	Malaria
Aged	2.70E‐01	14	79	EGFR tyrosine kinase inhibitor resistance
	2.70E‐01	20	129	Ribosome
	2.70E‐01	13	70	B cell receptor signaling pathway
	3.70E‐01	15	93	Endocrine resistance
	5.30E‐01	17	131	Oxidative phosphorylation
	5.30E‐01	12	84	ErbB signaling pathway
	5.30E‐01	18	137	Upiquitin mediated proteolysis
	5.30E‐01	16	123	Osteoclast differentiation
	5.30E‐01	15	101	T cell receptor signaling pathway
	5.30E‐01	27	227	Thermogenesis

**FIGURE 6:**
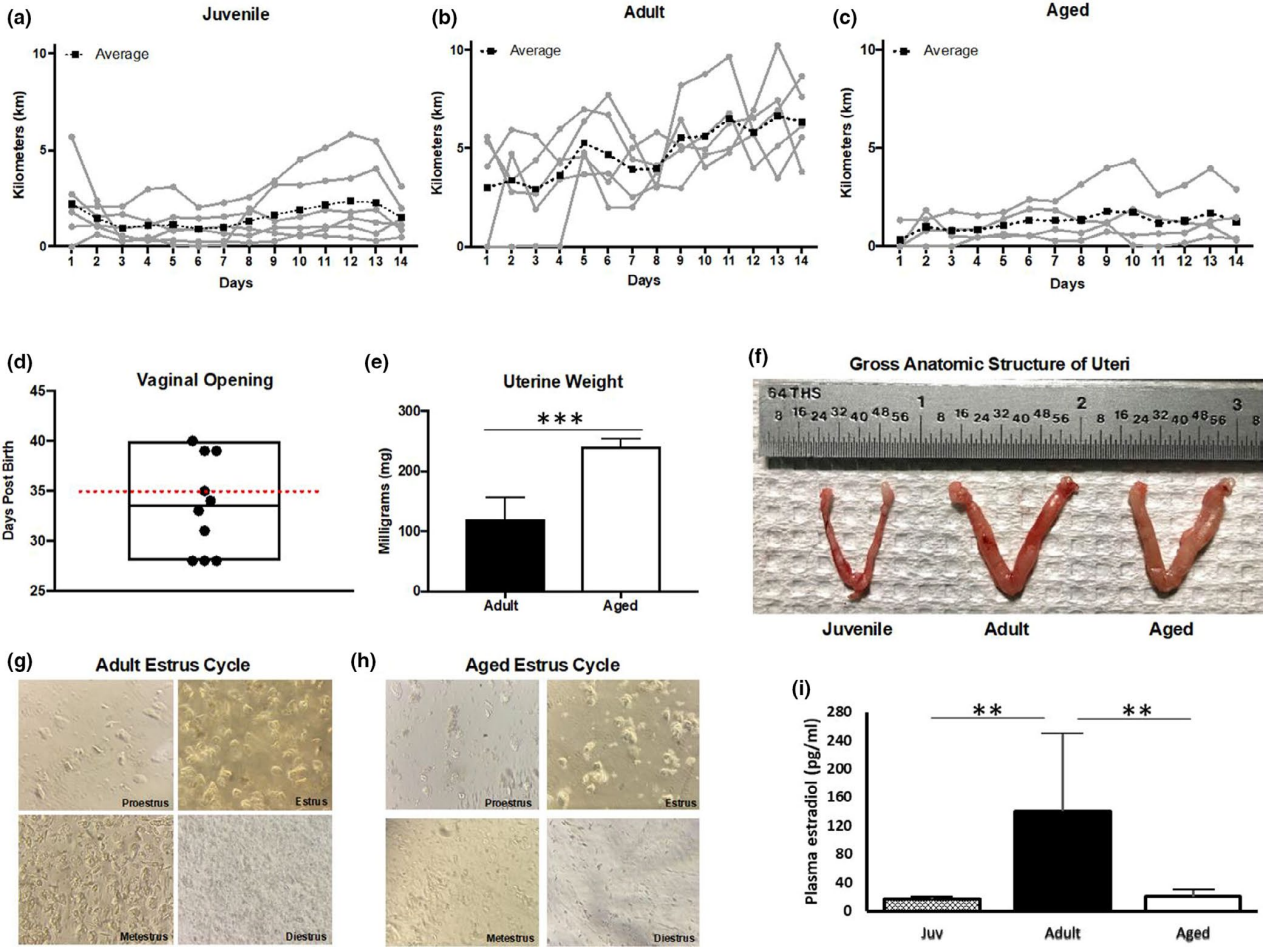
Analysis of sexual maturity and reproductive competence in juvenile, adult, and aged female mice. (a‐c) Daily running distance for juvenile, adult, and aged female mice. Each gray line is an individual female mouse with the black dotted line representing the average (n = 6). (d) Vaginal openings for juvenile mice (n = 10), Red dotted line on day 35 post‐birth indicates the age at euthanasia; (e) Uterine weight for adult (n = 8) and aged (n = 6) mice. ***p < 0.001 by Student's t‐test; (f) Representative images of a juvenile, adult, and aged uterus; G‐H) representation of estrous cycling in adult and aged female mice; (i) female plasma estradiol levels across the life course, n = 5 per group **p < 0.01 by one‐way ANOVA

Given the historical assumption that sex differences are attributed to estrogen that significantly changes across the life course (Nugent et al., 2012), we aimed to indirectly quantify estrogen through the sexual maturity and reproductive competence of our female mice. Sexually mature female mice demonstrate cyclicity in wheel running that is regulated by estrogen (Aguiar et al., 2018), thus we monitored wheel‐running activity. Juveniles did not show a cyclic pattern for wheel running (Figure 6a), but sexually mature adult females showed cyclic patterns over 14 days (Figure 6b). Aged mice did not show a cyclic wheel‐running pattern (Figure 6c), except one mouse that had a similar pattern to the juveniles. To identify the sexual maturity of juvenile mice, we assessed vaginal opening which occurred at 33.5 ±1.5 days with the earliest vaginal opening detected at 28 and the latest at 40 days (Figure 6d). Uterine weight has been suggested as a surrogate for estrogen (Lindberg et al., 2002) and in our hands, uterine weight was ~2‐fold higher in aged compared to adult mice (Figure 6e,f). Further supporting reproductive incompetence in aged mice, we performed vaginal cytology in adult and aged mice (Figure 6g,h). Unlike adults which demonstrated the anticipated 4‐day estrus cycle, it was challenging to find aged mice in the estrus phase and diestrus in aged females was less pronounced than in adults. To link these indirect measures of estrogen to direct estrogen concentrations, we quantified plasma 17β‐estradiol. As expected, plasma concentrations were highest in adults which were also the most variable given our lack of control for the estrous cycle, with significantly lower concentrations in juvenile and aged mice (Figure 6i). Together, these data suggest that estrogen alone is unlikely to explain the differences in female gene expression across the life course, given that cardiac transcriptomics and sex differences could not be explained by differences in estrogen levels.

### 3.4 Plasma proteomics across the male life course

**FIGURE 7:**
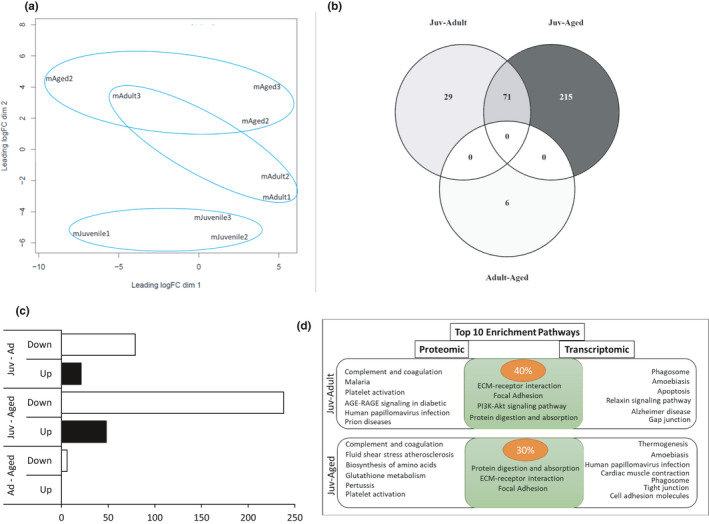
Differentially Expressed Proteins (DEP) in male plasma across the life course. (a) PCA plot for male age: juvenile (mJuvenile‐ male juvenile), adult (mAdult – male adult), and aged (mAged‐ male aged); (b) Venn diagram for differentially expressed proteins in males (FDR 5%): Juv‐Adult, adult compared to juvenile as a baseline; Juv‐Aged, aged compared to juvenile as a baseline; adult‐aged, aged compared to adult as a baseline; (c) Bar graph for up‐ and downregulated DEPs; Juv‐Ad, DEPs from adult compared to juvenile as a baseline; Juv‐Aged, DEPs from aged compared to juvenile as a baseline; Ad‐Aged, DEPs from aged compared to adult as a baseline. (d) Diagram of overlapping pathways from top 10 enriched pathways from proteomic and transcriptomic analyses for Juv‐Ad and Juv‐Aged comparisons. n = 3 per group; FDR < 0.05

To understand the impact of circulatory factors on cardiac aging and determine if circulating factors correlated with changes in cardiac gene expression, we performed label‐free quantification of the plasma proteome in male mice. PCA showed clustering and distancing of the three ages (Figure 7a). Comparison of juvenile‐adult plasma proteome produced 100 differentially expressed proteins (DEP): 79 downregulated and 21 upregulated. Juvenile‐aged plasma proteome comparison produced 286 DEP: 238 downregulated and 48 upregulated. Similar to RNA‐seq, plasma proteomic analysis of adult‐aged produced a limited number of DEP, all downregulated with age (Figure 7b,c). Juvenile‐adult DEP were clustered mostly in ECM organization and immune response activation pathways (Table 3). Juvenile‐aged DEPs were clustered in metabolic pathways, ECM organization, and immune response activation pathways (Table 3). Since adult‐aged comparison had only six DEPs, gene ontology enrichment analysis did not produce any results.

**TABLE 3:** Gene Ontology Enrichment Analysis for DEPs in male plasma protein across the life course (FDR 5%)

	Males			
	Enrichment FDR	Genes in list	Total genes	Functional Category
Juvenile‐Adult	8.26E‐07	7	83	ECM‐receptor interaction
	2.69E‐04	5	88	Complement and coagulation cascades
	9.46E‐04	6	198	Focal adhesion
	2.28E‐03	4	90	Protein digestion and absorption
	3.41E‐03	3	47	Malaria
	4.88E‐03	4	123	Platelet activation
	7.19E‐03	6	353	PI3K‐Akt signaling pathway
	7.19E‐03	6	348	Human papillomavirus infection
	1.44E‐02	3	101	AGE‐RAGE signaling pathway in diabetic complications
	1.44E‐02	2	34	Prion diseases
Juvenile‐Aged	5.72E‐13	16	88	Complement and coagulation cascades
	5.27E‐05	11	142	Fluid shear stress and atherosclerosis
	4.06E‐04	8	90	Protein digestion and absorption
	9.51E‐04	7	77	Biosynthesis of amino acids
	1.24E‐03	7	83	ECM‐receptor interaction
	1.94E‐03	6	64	Glutathione metabolism
	1.94E‐03	10	198	Focal adhesion
	3.86E‐03	6	76	Pertussis
	7.95E‐03	7	123	Platelet activation
	9.40E‐03	4	36	African trypanosomiasis
Adult‐Aged	No Clustering			

## 4 DISCUSSION

The risk for heart disease increases with advanced age with distinct clinical outcomes between men and women. Identification of the sex‐specific mechanisms of cardiac aging is critical for the identification of therapies to slow cardiac aging and age‐related heart disease. In the present study, we performed RNA‐seq in cardiac tissue at three distinct ages in both male and female mice. Age‐related changes in cardiac gene expression differed significantly between males and females‐ both with respect to pathway enrichment and in terms of the temporal nature of these differences. Significant sex differences were also observed, with the largest differences in gene expression at the youngest and oldest ages. In contrast to traditional paradigms attributing sex differences to hormone‐based estrogen and estrous cycling in adult animals, transcriptomic variance peaked in sexually immature juveniles and reproductively incompetent aged mice. Given our previous reports of age‐related changes in cardiac function and structure (Yusifov et al., 2021), these findings of sex‐specific differences in cardiac gene expression across the life course are significant. Understanding these sex‐specific age‐related differences and how they contribute to increased risk for heart disease with advanced age is critical to slow cardiac aging.

### 4.1 Gene ontology pathways across the life course

The extracellular matrix‐receptor network plays a fundamental role in cardiac function, not only by providing structural support, but also by facilitating force transmission and signal transduction between cardiomyocytes and the extracellular environment (Theocharis et al., 2016). Changes in the ECM are implicit in cardiac aging as well as age‐related cardiac diseases such as dilated cardiomyopathy, ischemic heart failure, and hypertrophy (Frangogiannis, 2019; Gallagher et al., 2007). RNA‐seq identified a significant number of ECM‐related genes in juvenile‐adult (9 DEGs) in males, and in juvenile‐aged comparison (24 DEGs) in females, suggesting that while ECM changes occur with advanced age, they do so earlier in males compared to females. Further, females demonstrated lower COL1A1 expression in the aged heart, while males demonstrated lower expression in adulthood. These data are analogous to clinical studies where women demonstrate delayed cardiovascular aging compared to men (Coutinho, 2014; Merz & Cheng, 2016), as well as our previous work in these animals demonstrating sex differences in fibrosis in the aged heart (Yusifov et al., 2021). Thus, the aging mouse can be used to investigate the molecular mechanisms driving sexual dimorphisms in age‐associated cardiac fibrosis. Extrapolating changes in pro‐fibrotic gene expression to the deposition of fibrosis are challenging due to the complex regulation of the ECM. Age‐associated fibrosis has historically been thought to be due to increased expression of pro‐fibrotic factors, although the exact mechanism of this process is not clear. Understanding the mechanisms by which male and female hearts becomes fibrotic with advanced age is important—whether due to the elevated deposition of fibrotic proteins, lower turnover, and removal of said proteins, changes in post‐translational modification of collagen and fibrous proteins, or a combination thereof, is of enormous significance for cardiac aging.

Inflamm‐aging is defined by chronic elevated levels of inflammatory markers that carry high susceptibility to chronic morbidity, disability, frailty, and correlates with cardiac dysfunction (Baylis et al., 2013; Ferrucci & Fabbri, 2018). Consistent with this notion, our data show hallmarks of inflamm‐aging both in RNA‐seq as well as in proteomics. Similar to ECM gene expression, inflammation pathways do not appear in the enrichment of DEGs from female juvenile‐adult, but they do in males. This suggests early activation of inflammation in males compared to females, which has been previously defined as a sexual dimorphic pathway in human immune system aging (Márquez et al., 2020). There is increasing interest in developing novel therapies that target the immune response to improve cardiac repair following cardiac injury and our data add to the significant need to identify and develop these novel therapies by considering sex dimorphism as a major biological variable in cardiac immune health.

Metabolic changes in the heart contribute to the development and progression of cardiac dysfunction and age‐related cardiac disease. In some clinical studies, along with age‐related changes, sex differences in metabolism, both at baseline and with cardiac pathology have been reported (Kichuk‐Chrisant, 2002; Piquereau & Ventura‐Clapier, 2018). In line with these studies, our female RNA‐seq data show significant changes in metabolic pathways from juvenile to adulthood. However, male DEGs did not cluster in metabolic pathways across the life course, consistent with reports of sex differences in humans with respect to cardiac metabolism, with the adult female myocardium demonstrating a greater reliance on carbohydrate and glycolytic metabolism (Wittnich & Wallen, 1997). In pathological female hearts, these energy requirements have been suggested to relate to increased ventricular dysfunction due to the rapid accumulation of anaerobic end‐products with premature inhibition of glycolysis, rapid depletion of ATP, and decreased functional recovery (Parrish et al., 1987; Wittnich et al., 2007). Our findings on sex‐specific differences in the metabolic shift across the age may offer a potential direction to investigate molecular mechanisms behind these differences.

### 4.2 Transcriptomic differences by sex

Despite recent efforts to increase female representation in clinical and pre‐clinical trials, sex difference research is still in its infancy, as are investigations about how sex differences change across the life course (Khramtsova et al., 2019). Because gene expression in the LV might provide insights into potential mechanisms for sex‐specific differences in disease prevalence and progression, we also compared transcriptional profiles in male versus female mice within each age across the life course. In our dataset, the majority of DEGs by sex demonstrated absolute fold changes twofold and higher with greatest enrichment on sex‐linked chromosomes, as expected. However, not all DEGs were associated with sex chromosomes and enrichment of non‐sex linked genes and pathways suggested that transcriptomic differences in juvenile male‐females were different from aged male–females.

The estrus cycle influences many physiological processes (Kopp et al., 2006; Yang et al., 2006) and in healthy sexually mature adult females gives cyclic character to many of these processes. We expected to identify a significant number of differentially expressed genes in adult males versus females, based on our lack of control for the estrus cycle. However, we were surprised to find significant transcriptomic sex differences in juveniles and aged mice, with few DEG in adults. This finding is surprising given that the juvenile animals are sexually immature as evidenced by a lack of vaginal opening (Mayer et al., 2010) and the aged animals were reproductively incompetent, as evidenced by differences in uterine weight and cessation of estrus cycling (Han et al., 2013). Furthermore, daily voluntary wheel‐running patterns, known to correlate with cyclicity in estrogen (Aguiar et al., 2018), also supported that estrogen was not regulating physical activity in juvenile or aged animals, given that daily patterns did not demonstrate cyclicity as in adult females. Together with the plasma quantification of estrogen, the sex differences reported in the current work demonstrate that significant transcriptomic differences in juveniles and aged mice are not directly or simply due to estrogen and suggest that the targeted investigation of other potential biological factors will be necessary to understand the nature of these sex differences and with changes in female sex hormones across the life course.

### 4.3 The plasma proteome as a surrogate for cardiac gene expression

Although new Omics approaches provide a powerful tool for understanding biological processes in the heart, it has the disadvantage of requiring cardiac tissue samples both in clinical and research setting as well as acquiring pathological samples and normal healthy tissue, both of which are limited in the cardiac field. However, sample collection from circulating blood is convenient and does not require risky interventional procedures. Therefore, changes in circulation have been suggested as a future source of biomarkers for disease prognosis and diagnostics (Hanash et al., 2011). Therefore, we assessed plasma proteomics in our mice and interestingly found that the blood proteome differences by age mirrored our results from the LV transcriptome. Gene ontology analysis showed the clustering of DEP in functional categories similar to the transcriptome functional categories (Figure 7d). Although more work needs to be performed to extrapolate circulating factors to the heart, our work suggests that the plasma proteome can play a surrogate role for cardiac gene expression, which would enable the design of future interventions to understand or treat cardiac aging by the assessment of blood‐based methods, rather than cardiac‐specific analysis.

## 5 LIMITATIONS AND CONCLUSIONS

While our investigation included three distinct ages of mice, a novel experimental design compared to literature to date which at most only compares two ages, one of the limitations of this work is the omission of additional ages. We could benefit from the addition of a younger juvenile animal (2–3 weeks) which is developmentally immature, as well as an older animal around 24 months old. In addition, a middle‐age of around 12 months would be significant in the females, given the later trajectory of changes in cardiac gene expression, to begin to understand when these differences in gene expression begin to occur. These experiments were performed on animals housed in Laramie, WY, located at 7200 feet (2,190 m) above sea level. Given the well‐known impact of hypoxia on aging and on cardiac function, it is possible that our conclusions would be different if performed at sea level. We also recognize that one of the limitations of this work is the exclusion of plasma proteomics from female mice. Given how similar the plasma proteomics and cardiac transcriptomics were in the males, it is important to know if these similarities exist in female plasma as well. In conclusion, cardiac aging shows distinct trajectories in cardiac transcriptomic profiles between males and females, indicating fundamental sex differences and demonstrates the need for the consideration of age and sex as biological variables in heart disease.

## CONFLICT OF INTEREST

The authors declare no conflicts of interest.

## AUTHOR CONTRIBUTIONS

DRB and KCW conceived and designed the project. AY, EKK, CEW, and EES performed experiments and collected data. DRB, KCW, VEC, EES, and AY analyzed and interpreted data. AY and DRB drafted the manuscript. All authors reviewed and revised the manuscript, approved the final manuscript as submitted, and agreed to be accountable for all aspects of the work.
